# Hair Follicle Seedling Cryomicroneedles from Hierarchical Microfluidic Organoid on a Chip

**DOI:** 10.1002/advs.75961

**Published:** 2026-06-04

**Authors:** Xinyue Cao, Dongyu Xu, Minhui Lu, Yuanjin Zhao

**Affiliations:** ^1^ Department of Rheumatology and Immunology School of Biological Science and Medical Engineering Nanjing Drum Tower Hospital Southeast University Nanjing China; ^2^ Wenzhou Institute University of Chinese Academy of Sciences Wenzhou China

**Keywords:** hair follicle, hair regeneration, microfluidics, microneedle, organoid, organ‐on‐a‐chip

## Abstract

Hair follicle organoids (HFOs) have demonstrated revolutionary regenerative potential in hair follicle regeneration, while their high‐throughput production, uniform morphology, excellent hair growth potential, and simple and efficient intradermal transplantation are still challenges. In this paper, we present novel hair follicle seedling cryomicroneedles to achieve bionic hair regeneration by using a hierarchical microfluidic organoid‐on‐a‐chip. Our integrated chip features a fluidic channel module and a hierarchical microneedle template module, enabling microflow‐guided uniform cell distribution into needle‐shaped microwells to form HFOs. Cryopreservation agents (CPA)‐containing pregel solutions are injected to encapsulate HFOs following UV gelation, and the final HFO‐loaded cryomicroneedle (cryoMN@HFO) is obtained after freezing and demolding. We have demonstrated that the cryoMNs@HFOs could preserve homogeneous HFO morphology, favorable viability, and excellent hair growth potential. Besides, the cryoMN@HFOs possess good skin penetration ability and biosafety, enabling rapid transplantation of HFOs into the dermis. Thus, after intradermal transplantation in animal experiments, the delivered organoids develop into fully functional hair follicles with mature structures in vivo. Based on these advantages, we believe that this technology holds promise for human hair follicle reconstruction.

## Graphical abstract

In this paper, a hierarchical microfluidic chip is developed to produce uniform hair follicle organoids (HFOs) in cryomicroneedles. Cryopreservation agents encapsulate HFOs, preserving viability and hair growth potential. The cryomicroneedles enable simple intradermal transplantation with good skin penetration. In animal experiments, delivered organoids regenerate functional hair follicles, demonstrating promise for hair reconstruction.

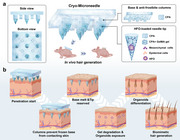

## 1 Introduction

Hair follicles (HFs), as unique mature organs in humans, have limited regenerative ability following atrophy or necrosis [1, 2]. Cutaneous scarring and alopecia‐related disorders that cause HF damage can result in irreversible hair loss and bring escalating psychological burdens to patients [3, 4, 5, 6]. Conventional treatments, including drug therapy and hair transplantation surgery, are essentially adjustments and redistribution of existing follicles rather than achieving regeneration of hair follicle donors, and therefore fail to address the problem of alopecia [7, 8, 9]. In contrast, hair follicle organoids (HFOs) derived from stem cell‐directed differentiation have shown revolutionary potential in HF regeneration [10, 11, 12, 13]. Following successful in vivo engraftment, these 3D HFOs can simulate the key characteristics of cyclic development of HFs, providing sufficient graft source for hair regeneration [14, 15, 16, 17]. Despite bringing new hope, current approaches fail to construct high‐throughput HFOs with uniform size and excellent hair growth potential. In addition, developing simple and efficient organoids' functional intradermal implantation methods to faithfully emulate the natural hair flow's appearance is another urgent challenge to be tackled. Given that, innovative strategies to construct and minimally invasive delivery of controllable HFOs are urgent to be developed.

**FIGURE 1:**
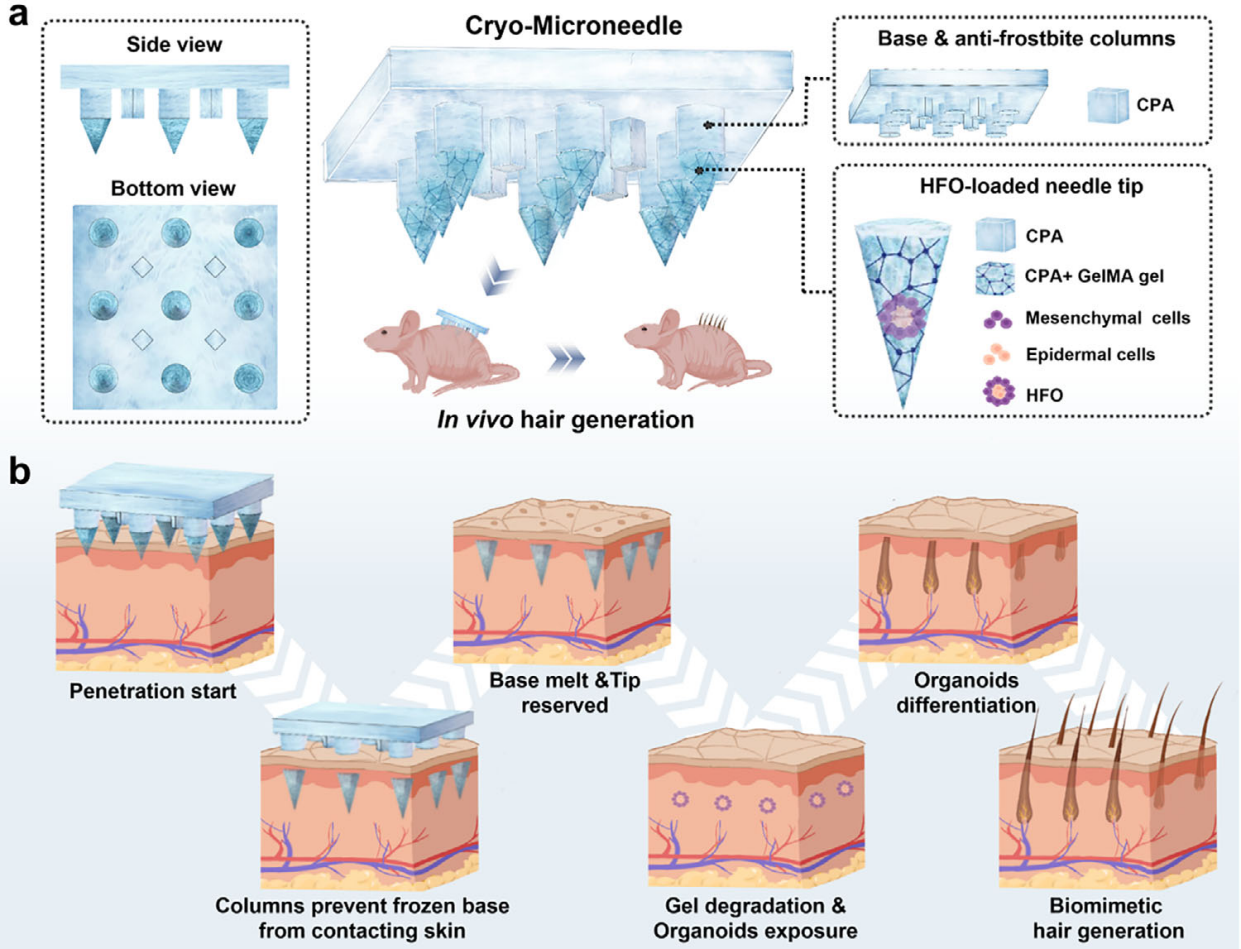
Schematic of (a) the composition of HFO‐loaded cryo‐microneedle (cryoMN@HFO) and (b) its application for promoting hair generation.

Herein, we proposed a hierarchical microfluidic HF organoid‐on‐a‐chip in response to the above‐mentioned challenges, as schemed in Figure 1. Microfluidic technology enables precise manipulation of fluids at the micrometer scale, where the meticulously designed multi‐channel networks facilitate efficient transport and accurate control of minute volumes of liquids [18, 19, 20, 21]. Integrated with the cultivation of cells and organoids, microfluidic organ‐on‐a‐chip systems can recapitulate the microenvironment, architecture, and function of human organs, thereby providing physiologically relevant in vitro models for drug screening and disease research [22, 23, 24, 25, 26, 27]. In contrast, hierarchical structures comprising multi‐scale ordered assemblies can exhibit some emergent functionalities through scale‐dependent interaction [28, 29, 30]. Notably, microneedles are typical advanced materials with hierarchical structures, consisting of the needle tips for skin penetration and a backing plate for mechanical stability. This structural design endows microneedles with great potential in transdermal delivery and precise drug administration [31, 32, 33, 34]. Therefore, we envisioned that the synergizing microfluidic spatial control and hierarchical microneedle delivery would create a novel organ‐on‐a‐chip for high‐throughput HFO culture and efficient intradermal transplantation for bionic hair regeneration.

In this paper, we constructed HFO‐loaded cryomicroneedles (cryoMN@HFO) from the hierarchical microfluidic organoid‐on‐a‐chip for HF reconstruction (Figure 1). The microfluidic chip, consisting of a fluidic channel module and a microneedle template module, was specifically designed. In the system, microfluidic flow was first employed to uniformly distribute embryonic epithelial and mesenchymal cells into needle‐shaped microwells, followed by the in situ production of HFOs with homogeneous morphology. After UV‐triggered polymerization, the HFOs could be encapsulated by injecting pregel solutions comprising cryopreservation agents (CPAs) and gelatin methacryloyl (GelMA) into the template's needles, utilizing coherent fluid exchange. The final cryoMN@HFO could be obtained after gradient freezing and demolding. As the frozen cryoMNs had robust transdermal delivery ability, they could rapidly deliver the organoids to the dermis. Notably, the backing plate with a specialized microcolumn design could act as an anti‐frostbite device to prevent direct contact between the skin and the frozen base. Following the functional assessment in vitro, the HFOs encapsulated in the cryoMNs have been demonstrated to maintain favorable viability and excellent hair growth potential. In further animal experiments, both the backing plate and needle tips of cryoMNs melted after transplanting without causing permanent skin damage. Attractively, the delivered homogeneous HFOs underwent a development process in the skin that resulted in the rebuilding of mature, completely functional HFs. Therefore, we believe that this cryoMN@HFO fabricated from hierarchical microfluidic organoid‐on‐a‐chip possesses great potential as a high‐throughput HFOs transplantation strategy for functional HF reconstruction.

## 2 Result and Discussion

**FIGURE 2:**
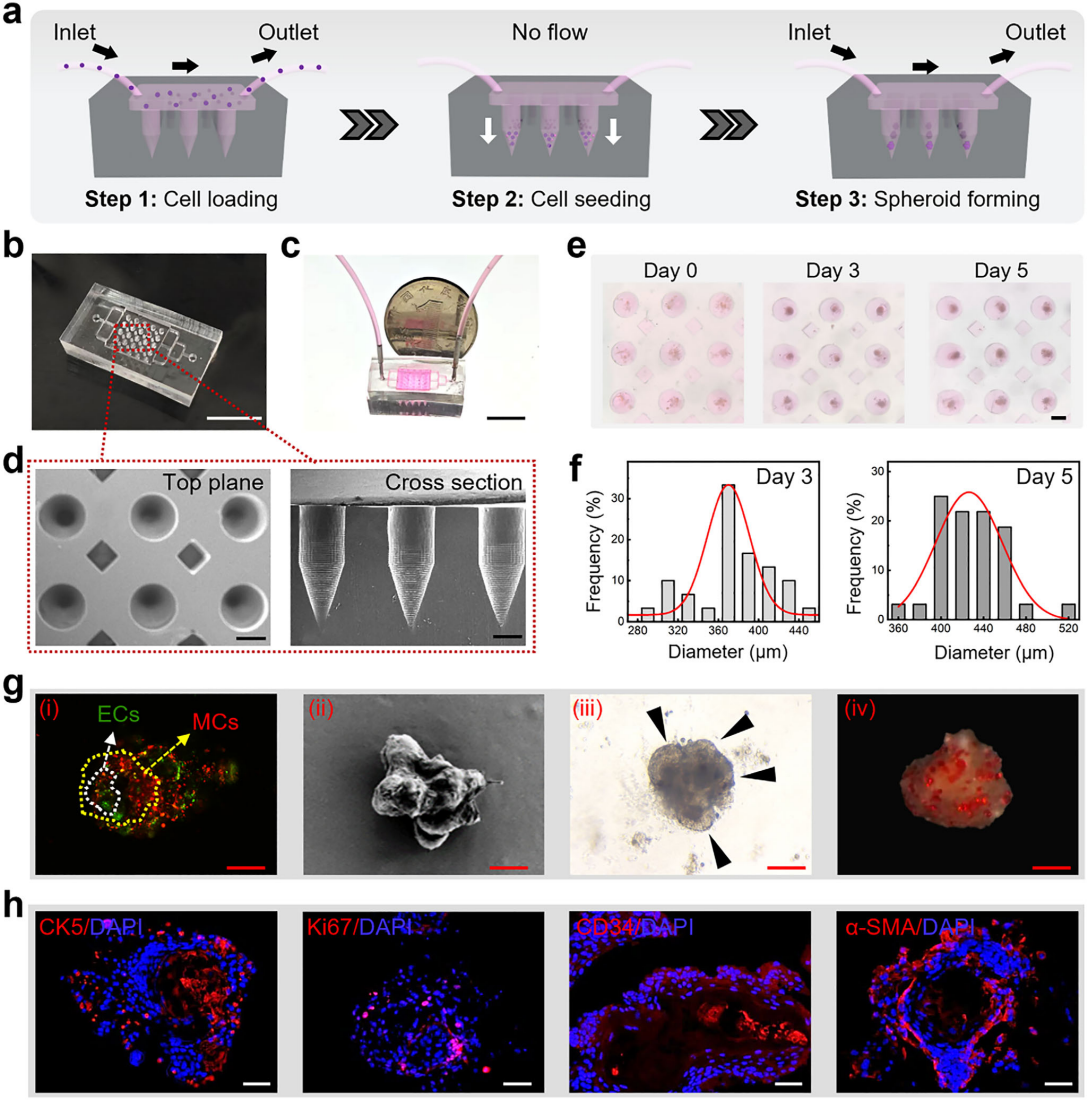
HFO Formation in the hierarchical microfluidic chip. (a) Schematic of HFOs' formation process, including cell loading, cell seeding, and HFO spheroid formation. (b, c) Optical images of the hierarchical microfluidic chip. Scale bars are 1 cm. (d) Scanning electron microscopy (SEM) images of the microwells arranged in the microfluidic chip's microneedle template. Scale bars are 500 µm. (e) Representative optical images of the HFOs' formation process in the microwells. Scale bar is 500 µm. (f) Diameter distributions of the formed HFOs in the microfluidic chip. (g) Analysis of HFOs. (i) Confocal laser microscope image; (ii) SEM image; (iii) Optical image; (iv) Oil red O staining result. Scale bars are 200 µm. (h) Immunofluorescence staining of typical cellular markers, including CK5, Ki67, CD34, and α‐SMA. Scale bars are 50 µm.

In this study, a customized hierarchical microfluidic chip, comprising a fluidic channel module and a microneedle template module, was designed to facilitate the perfusion of hair follicle stem cells and enable in situ generation of HFO spheroids (Figure 2a). Poly(dimethylsiloxane) (PDMS) was used to fabricate the chip with an overall size of approximately 2 cm^3^. It incorporated inlet and outlet channels at both ends to allow fluid exchange. The specialized microneedle template module consisted of a 5 × 5 array of conical micropores (800 µm in diameter, 1200 µm in height) connected at the base to a corresponding 5 × 5 array of cylindrical microcolumns (800 µm in diameter, 1000 µm in height). Additionally, a quadrangular prism‐shaped channel (300 µm in base length, 1000 µm in height) was integrated at the center of every 2 × 2 microcolumns (Figure 2b–d). Notably, this configuration also provided anti‐frostbite functionality. When cryogenic microneedles replicated from this template were applied to an agar‐based skin model, the needle tips penetrated the artificial skin, while the microcolumn arrays limited further insertion, thereby preventing extensive contact between the frozen backing plate and the skin (Figure S1). Consequently, this anti‐frostbite mechanism demonstrates promising potential for preventing frostbite injury in cryogenic microneedle applications through the hierarchical design, offering an innovative structural strategy to enhance biosafety.

During the fabrication of the specially made hierarchical microfluidic chip, its surface was modified with the hydrophobic polymer F127 to reduce pre‐perfusion cell adhesion, which is essential for the development of HFO spheroids. It should be noted that in conventional plate culture methods, the resulting cell spheroids often exhibit significant variations in size and morphology, leading to uneven transport of nutrients and oxygen. In contrast, our presented unique microfluidic chip demonstrates notable advantages in generating stem cell spheroids with uniform morphology while effectively reducing cell loss, thereby overcoming limitations of existing methods [35]. The chip enables precise control of the microflow of cell suspension, allowing embryonic epithelial cells (ECs) and embryonic mesenchymal cells (MCs) to be evenly distributed into individual microwells. Subsequently, the cells aggregated at the bottom of the microwells and gradually formed spheroids in a low‐adhesion environment (Figure 2e). Notably, cell aggregates generated from different initial cell numbers all exhibited highly consistent morphological characteristics. The diameter of spheroids formed by Day 5 also increased proportionally with the number of seeded cells: spheroids derived from about 2 × 10^4^, 4 × 10^4^, and 8 × 10^4^ cells had diameters of approximately 400, 440, and 510 µm, respectively (Figure 2f, Figure S2). Furthermore, the in vivo hair‐forming capacity of the constructed HFOs was assessed using a reported superficial wound model [36, 37]. The results indicated that both the hair‐forming ability and efficiency of HFOs improved with increasing cell number (Figure S3). Based on these findings, to ensure robust hair‐forming potential and match the microwell dimensions, a total of 4 × 10^4^ cells (comprising ECs and MCs at a 1:1 ratio) were selected for subsequent experiments. Under this condition, cell spheroids began to form on Day 3 and continued to grow, reaching a diameter of approximately 440 µm by Day 5, as shown in Figure 2e,f.

Notably, in studies related to HF and skin organoids, it has been reported that supplementation with a low concentration of Matrigel promotes cellular self‐organization. According to Fukuda et al., under Matrigel‐supplemented culture conditions, the two cell types (ECs and DCs at a ratio of 1:1) spontaneously form a core‐shell‐structured aggregate [15]. Given that, Vybrant DiO‐labeled ECs and Vybrant DiD‐labeled MCs were first prepared in our experiments (Figure S4). By day 5, the expected cellular aggregate structure was successfully observed, with ECs (green) clustered at the core surrounded by MCs (red) (Figure 2g (i)). Further morphological and histological analyses were performed on the HFOs cultured for 5 days. Scanning electron microscopy (SEM) image revealed prominent protrusions on the surface of individual aggregate (Figure 2g (ii)). Optical image showed that these protruding regions exhibited multiple pigmented areas, from which future HFs and shafts are expected to emerge (Figure 2g (iii)). Additionally, typical cellular markers of HFs and surrounding tissues were detected in the generated HFOs, including the adipose tissue marker Oil Red O (Figure 2g (iv)), the arrector pili muscle marker α‐smooth muscle actin (α‐SMA), the hair follicle stem cell marker CD34, the basal keratinocyte marker CK5, and the proliferating cell (hair matrix cell) marker Ki67 (Figure 2h). In summary, by introducing Matrigel‐containing medium, this microfluidic system enabled uniform distribution of the two cell types within needle‐shaped microwells and facilitated the in situ generation of HFOs with uniform morphology and typical hair follicle marker expression within 5 days.

**FIGURE 3:**
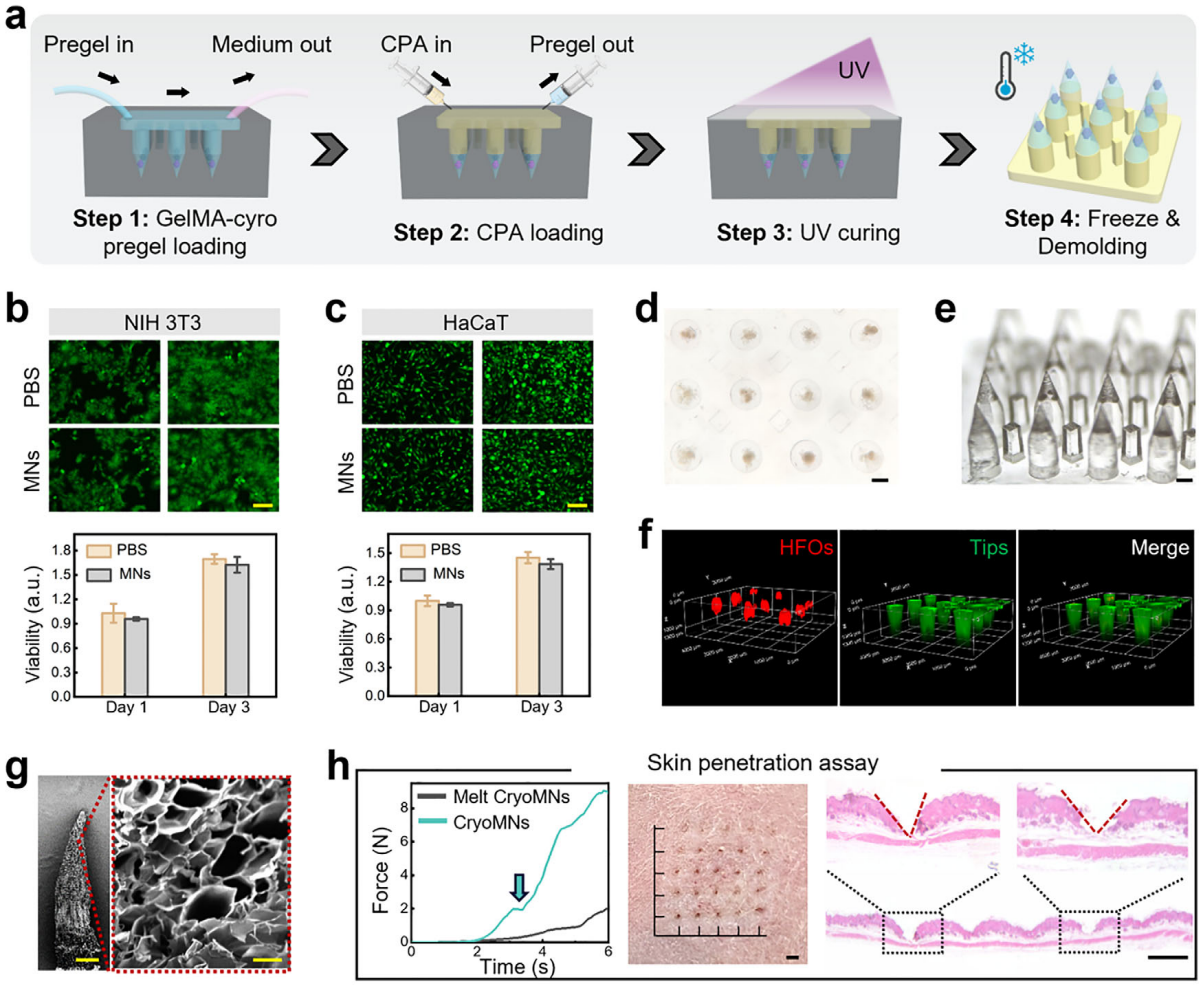
Fabrication and characteristics of cryoMN@HFO. (a) Schematic of cryoMN@HFO fabrication processes. (b, c) Representative fluorescence images and statistical analysis of cell viability assays in (b) NIH 3T3 and (c) HaCaT co‐cultured with melt cryoMNs. Scale bars are 200 µm. (d) Optical image of the HFOs in microwells after medium exchange. Scale bar is 500 µm. (e) Optical image of the cryoMN@HFO. Scale bar is 500 µm. (f) 3D reconstruction images of HFO distribution in microneedle tips. (g) Representative SEM images of a single microneedle tip. Scale bars are 500 µm (left) and 100 µm (right). (h) Evaluation of mechanical strength and transdermal ability. Scale bars are 1 mm. Of note, for the purpose of observing the morphology of the microneedle array (shown in Figure 3e–g), the pure CPA in the backing layer was temporarily replaced with GelMA‐cryo hydrogel to ensure that the base would not melt during observation.

The subsequent research objective is to achieve convenient and efficient transplantation of the constructed HFOs into the skin. In the field of transdermal drug delivery, cryoMNs fabricated using low‐concentration hydrogel materials demonstrate significant advantages in demolding performance, mechanical strength, and transdermal capability compared to conventional non‐frozen microneedles. Particularly in the context of live cell delivery, existing studies have confirmed that combining commercial CPA with GelMA hydrogel to form GelMA‐cryo hydrogels enables the construction of microneedles with structural integrity and reduced tip breakage [38]. Furthermore, dimethyl sulfoxide (DMSO) present in the CPA can effectively mitigate cell damage caused by ice crystal formation during the subsequent freezing process. As outlined in the overall process in Figure 3a, after successfully obtaining well‐formed HFOs, a GelMA‐cryo pregel solution containing 10% (w/v) GelMA mixed equally with CPA (containing 10% DMSO) was introduced into the microfluidic chip, and the culture medium was completely removed. The chip cover was then removed, and the pregel solution in the base and column array region of the hierarchical microneedles was replaced with pure CPA solution. Following UV irradiation for tip crosslinking, a programmed freezing process was applied, ultimately resulting in the detachment of the cryoMN@HFO from the PDMS mold. Notably, the resulting cryoMN exhibited a detachable backing layer characteristic: upon skin insertion, the frozen backing layer (composed of pure CPA) would liquefy, thus leaving only the crosslinked GelMA‐cryo hydrogel microneedle tips within the tissue.

It is important to emphasize the biosafety of the cryoMNs since they are intended for intradermal transplantation. Therefore, we systematically evaluated the cytocompatibility and hemocompatibility of the cryoMNs. Two model cell types (NIH 3T3 and HaCaT) co‐cultured with the melt cryoMNs retained normal cell morphology and viability, similar to the control group, as seen in Figure 3b,c. Furthermore, the material showed a low hemolysis rate (< 5%), suggesting that the cryoMN's hydrogel system possesses favorable biosafety and is non‐hemolytic (Figure S5). In this study, to facilitate morphological observation of the cryoMNs, the pure CPA in the base section was replaced with GelMA‐cryo hydrogel to enable the microneedles to maintain a normal structure after lyophilization. Following freezing and demolding, a typical cryoMN@HFO patch was successfully fabricated. Optical microscopy images revealed well‐defined needle tips, each containing a single HFO (Figure 3d–f). Further evaluations were conducted on the scaffold structure and mechanical properties of the GelMA‐based cryoMNs loaded with HFOs following vacuum freeze‐drying. As shown in the SEM images in Figure 3g and Figure S6, the lyophilized GelMA cryoMNs loaded with organoids exhibited a well‐preserved scaffold structure with uniform dimensions and interconnected micropores. In addition to facilitating cell attachment and offering an increased surface area for cell migration and proliferation, this interconnected porous network of the needle tip also supported intercellular signaling, waste elimination, and nutrient diffusion. More importantly, regarding mechanical strength and transdermal capability, we observed that even after the loss of reinforcement provided by ice crystals, the photochemically crosslinked GelMA hydrogel maintained structural integrity. To compare the mechanical properties of the microneedles in frozen versus melted states, both frozen and melted cryoMNs were subjected to mechanical testing. The force‐time curves were presented in Figure 3h. The frozen cryoMNs exhibited a smooth curve and withstood a load of 1.815 N per needle tip, indicating no sudden fracture of the tips. Particularly, this amount of force is sufficient for skin penetration (required range: 0.1–3 N) [38]. In contrast, the melted cryoMN@HFO nearly completely lost mechanical strength. Leveraging the mechanical strength of the cryoMNs, the organoids could be successfully delivered into the mouse dermis. A 5 × 5 array of microneedle penetration sites was clearly observed both macroscopically and in hematoxylin and eosin (H&E)‐stained frozen sections (Figure 3h).

**FIGURE 4:**
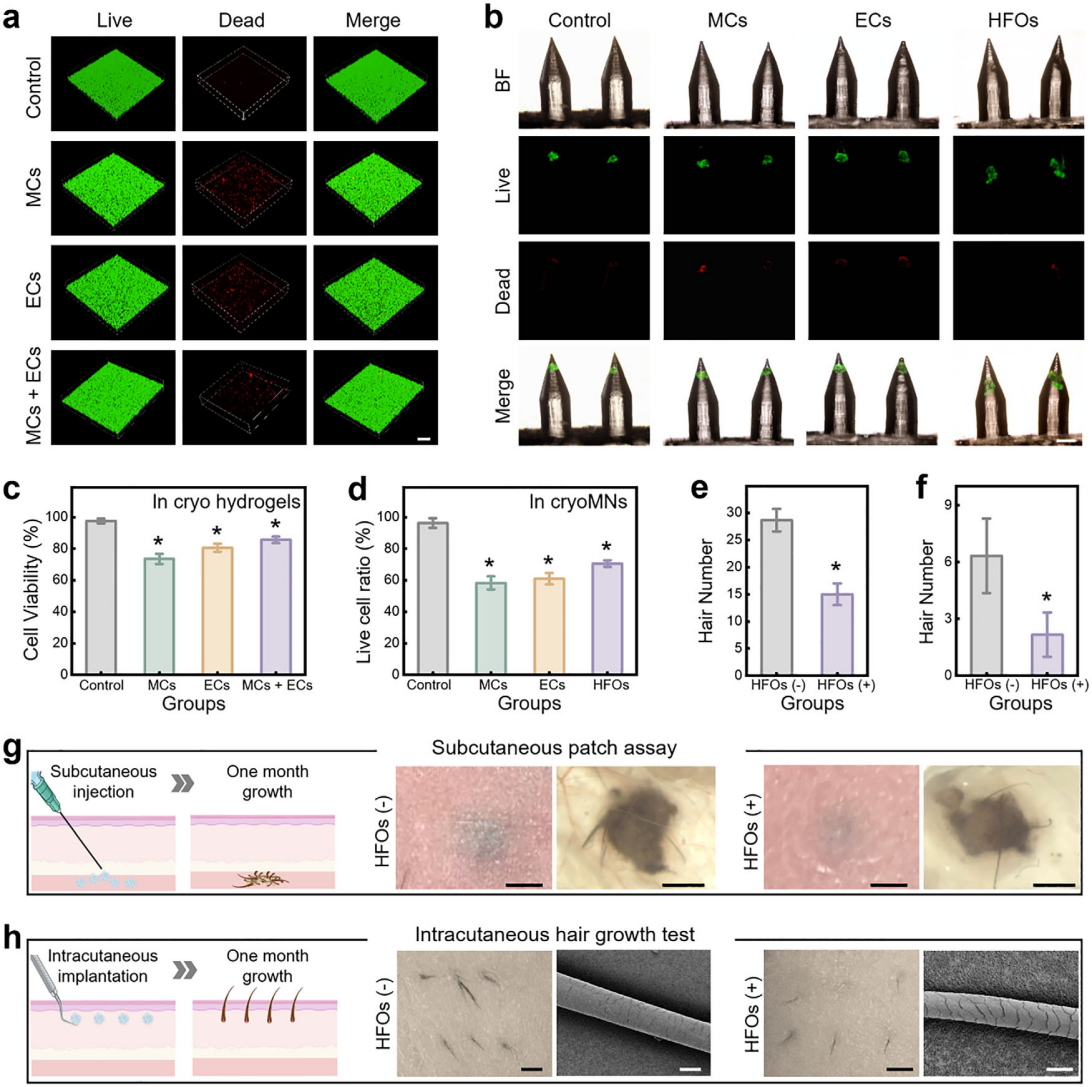
Test of cell viability and hair‐forming potential. (a) Live (green)/dead (red) cell staining of MCs, ECs, and their mixture (1:1 ratio) in GelMA‐cryo hydrogel under sequential freezing. Scale bar is 1 mm. (b) Live (green)/dead (red) cell staining of MC spheroids, EC spheroids, and HFO in cryoMNs under sequential freezing. Scale bar is 500 µm. Here, for the purpose of observing the microneedle array's morphology and cell spheroid distribution, the pure CPA in the backing layer was temporarily replaced with GelMA‐cryo hydrogel to ensure that the base would not melt during observation. (c) Statistical analysis of live/dead cell staining results in (a). (d) Statistical analysis of live cell ratio in (b). (e, f) Statistical analysis of the hair formation ability of HFOs before (HFOs (‐)) and after (HFO (+)) sequential freezing in the subcutaneous patch assay (e) and the intracutaneous hair growth test (f). (g) Schematic and result images of the subcutaneous patch assay. Scale bars are 5 mm. (h) Schematic and result images of the intracutaneous hair growth test. Scale bars are 2 mm in optical images and 20 µm in SEM images. Not significant (ns, p>0.05), ^*^ *p* ≤ 0.05, ^**^ *p* ≤ 0.01, and ^***^ *p* ≤ 0.001.

After confirming that the prepared cryoMNs exhibited satisfactory biosafety, expected scaffold structure, and sufficient mechanical strength, we further evaluated the cryoprotective effect of the CPA on cell viability. Initially, ECs or/and MCs were encapsulated in UV‐polymerized GelMA‐cryo hydrogel patch and subjected to a programmed freezing process. Live/dead staining results indicated that both MCs and ECs maintained high viability after freezing and subsequent recovery at 37°C in the GelMA‐cryo hydrogel. The cell viability rates were 73.67% ± 1.52% for MCs alone, 80.08% ± 2.51% for ECs alone, and 85.65% ± 2.08% for the MCs/ECs mixture at a 1:1 ratio (Figure 4a,c). In contrast, conventional GelMA hydrogel without CPA showed notably higher cell mortality, with less than 20% of cells in the mixed group surviving after freeze‐thaw recovery (Figure S7). Subsequently, cryoMNs were fabricated using the above formulations, and cell viability within the needles was examined again. The tip portion of the microneedles fabricated from the programmed freezing approach maintained good cellular viability. There were a few dead cells scattered throughout, but no notable necrotic cell clusters were observed. Data analysis revealed that the overall cell viability in CPA‐containing cryoMNs reached 70.64% ± 2.14%, consistent with earlier findings (Figure 4b,d). In summary, these results showed that the two types of cells constituting the HFOs have been successfully cryopreserved within the GelMA‐cryo hydrogel's 3D matrix and endured difficulties like UV radiation, low temperatures, and mechanical processes.

In addition to cell viability, we also evaluated whether the HFOs retained their HF‐forming potential after undergoing programmed freezing. A classical hair patch assay was first performed to assess HF reconstruction (Figure 4g). Both non‐frozen and cryo‐preserved HFOs (5 aggregates each) were subcutaneously injected into the lateral dorsal skin of nude mice using a standard injection needle. At 28 days post‐transplantation, obvious pigmentation was observed at the transplant sites, although no hair shafts had yet broken through the skin surface. After one month, a clearer view from the underside of the removed skin revealed that black hair shafts had grown at every transplanted site. After the tissue was enzymatically dissociated, the clusters of newly formed hair shafts were harvested, and the total quantity of hairs at each location was counted (Figure 4e). Although the hair‐forming capacity of frozen HFOs was significantly lower than that of fresh HFOs, both groups produced more than 10 hairs per site, indicating that cryopreserved HFOs still retained considerable hair‐inducing ability. In addition to the conventional patch assay, we further conducted in vivo experiments to evaluate the *de novo* HF formation ability of HFOs before and after freezing (Figure 4h, f). Here, a 20‐gauge ophthalmic V‐Lance knife was used to make superficial stab wounds on the backs of nude mice. Both kinds of HFOs (before and after freezing) were then transplanted into the wounds, with one aggregate per wound. The transplantation areas were observed weekly, and photos of newly grown hair were recorded. By four weeks after transplantation, the normal HFOs had produced abundant black hair, while frozen HFOs showed reduced but still detectable hair growth, consistent with the above patch assay results. Moreover, the presence of hair cuticle layers and other typical morphological characteristics was verified by SEM images of the hair shafts produced in both groups. It is noteworthy that each individual HFO yielded more than one HF in both experiments. This is attributed to the organoids' ability to form outward protrusions in multiple directions and develop pigmented regions, from which new HFs emerge. This observation has been further confirmed through long‐term in vitro culture (Figure S8). In conclusion, HFOs still retained significant hair‐generating capacity after programmed freezing, supporting their potential use in combination with cryoMN‐based transdermal delivery strategies.

**FIGURE 5:**
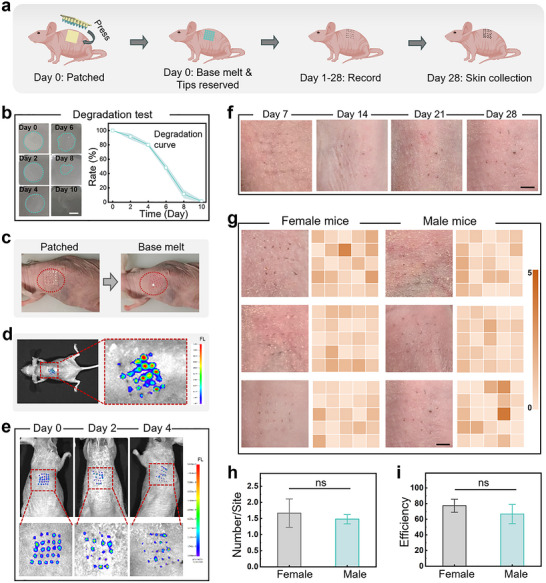
In vivo HF formation evaluation of the cryoMN@HFO. (a) Schematic description of animal experiments. (b) Images and statistical analysis of the hydrogel degradation process. Scale bar is 1 cm. (c) Photographs of the application of cryoMN@HFO onto nude mouse skin. (d) In vivo fluorescent imaging of nude mice treated with cryoMN@HFO. “FL” is fluorescence. (e) In vivo fluorescent imaging of the implanted HFOs in the mouse skin on day 0, 2, and 4. (f) Application of the cryoMN@HFO to the mouse dorsal skin at day 7, 14, 21, and 28 in vertical view. Scale bar is 2 mm. (g) Photographs of all delivery sites with cryoMN@HFO in different sexually nude mice at day 28. Scale bar is 2 mm. (h, i) Statistical analysis of (h) formed hair number (per site) and (i) hair formation efficiency in different sexual nude mice at day 28.

Building upon the convenience offered by the customized cryoMNs and the excellent hair‐forming capacity of HFOs, we further conducted in vivo experiments to evaluate the potential of cryoMN@HFO to reconstruct biomimetic HFs with normal physiological structures. As outlined in Figure 5a, cryoMN@HFO patches were prepared to be applied to the dorsal skin of nude mice. Notably, the degradation behavior of the hydrogel tips was first examined to verify whether the HFOs could be properly exposed post‐transplantation to participate in HF regeneration. As shown in Figure 5b, GelMA‐cryo hydrogel films (2 cm in diameter, 5 mm in height) were completely degraded within one week in simulated body fluid, demonstrating rapid enzymatic dissolution that facilitates timely HFO exposure and subsequent differentiation. Upon application, the pure CPA‐based backing plate dissolved after administration, leaving only the HFO‐laden tips within the skin (Figure 5c). Photos of the transplantation sites were recorded weekly over the following month, and skin samples were collected on day 28 for further analysis. In a typical experiment, DiD‐labeled HFOs were loaded into cryoMNs and delivered to the mice's mid‐dorsal skin. The microneedle's base subsequently dissolved, enabling the successful implantation of the HFOs into the skin. Notably, well‐aligned fluorescent signals from the DiD‐labeled HFO arrays were clearly observed within the skin, confirming accurate and successful implantation (Figure 5d). These fluorescent signals remained detectable for several days afterward, validating the stability of the implant (Figure 5e).

By day 7 post‐implantation, all 25 implantation sites had brownish‐black pigmentation, while no noticeable injuries were visible on the skin's surface. Besides, little hair tufts started to appear on the skin by day 14, and the pigmentation had deepened even further. By day 21, distinct hair shafts were clearly observable, which eventually grew to approximately 2 mm in length by day 28 (Figure 5f). These results demonstrate that GelMA‐based cryoMN‐delivered organoids can successfully facilitate HF reconstruction and promote hair regeneration. Moreover, to further assess the influence of sex on the in vivo hair‐forming efficiency of HFOs, identical procedures were performed using three male and three female nude mice. Representative images of hair growth and related statistical results are shown in Figure 5g and Figure S9. Across all six animals (each with 25 implantation sites), over 70% of the sites achieved successful HF reconstruction, with obvious hairs penetrating the skin surface. Here, blank cryoMNs without HFOs loading were set as the control group (Figure S10). Of note, a potential explanation for of the failed hair growth at a few sites includes the mechanical failure of the needle tip during implantation or the compromised bioactivity of HFOs. More importantly, it is worth emphasizing that no significant difference in hair regeneration outcomes was observed between male and female mice (Figure 5h,i). In conclusion, the GelMA cryoMN system developed in this study enables precise control over the location and number of regenerated hairs, demonstrating excellent spatial controllability and reproducibility.

**FIGURE 6:**
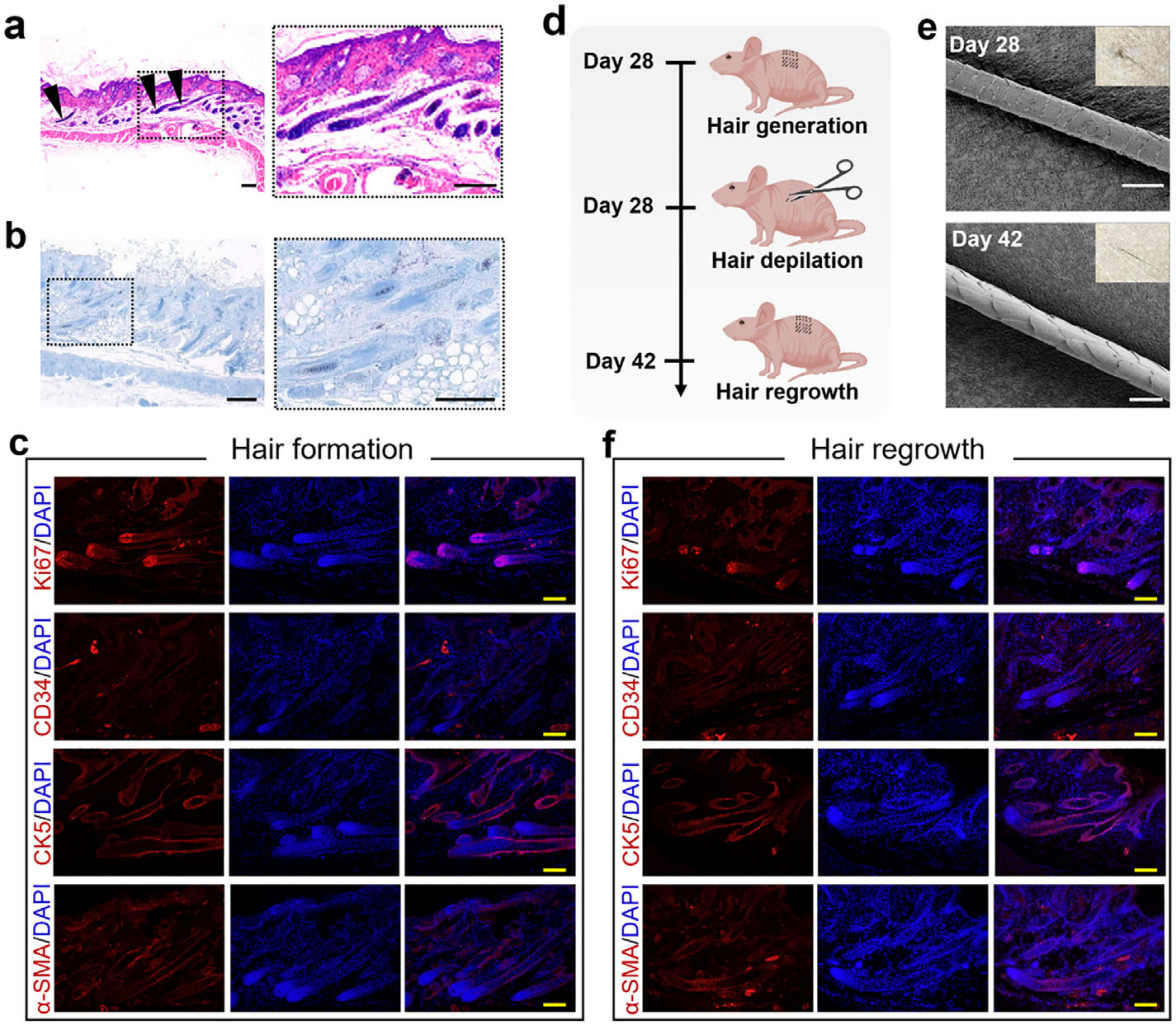
Molecular and structural validation of constructed HFs. (a) H&E staining images of the skin section with constructed HFs. Scale bars are 200 µm. The black arrow means constructed HFs. (b) Images of melanin‐containing HFs. Scale bars are 200 µm. (c) Immunofluorescence staining results of the constructed HFs on day 28. Scale bars are 200 µm. (d) Schematic illustration of the process of the depilation experiment. (e) Optical and SEM images of constructed HFs on day 28 (before depilation experiment) and reconstructed HFs on day 42 (after depilation experiment). (f) Immunofluorescence staining results of reconstructed HFs on day 42 (hair regrowth after depilation experiment). Scale bars are 200 µm.

On day 28, skin samples were collected and subjected to relevant histological analyses to more intuitively visualize the detailed structure of the regenerated HFs. Initially, recreating HFs with normal structural integrity and physiological function requires full structural organization and appropriate stratification. The HFO‐constructed HFs and the natural HFs in nude mice showed clear morphological changes on H&E staining of skin sections collected 28 days post‐transplantation with cryoMNs (Figure 6a). The hair shafts from the artificial HFs were seen to vertically pierce the stratum corneum from the dermis, just like the typical dorsal hair of C57BL/6J mice. Vacuolated loose connective tissue encircled the root sheath structures, and optical microscopy revealed the presence of melanin (Figure 6b). Beyond morphological integrity, a fully functional HF must also express specific marker proteins characteristic of its cellular components. To further molecularly and structurally verify the reconstructed HFs, a series of immunofluorescence staining analyses was performed to assess their reproducibility to the native phenotype and multi‐layered cellular architecture. As shown in Figure 6c, key molecular markers of various follicular compartments were systematically examined: these included the proliferation marker Ki67, mainly localized in the hair matrix region, indicating active cell proliferation; the bulge stem cell marker CD34, identifying regions enriched with regenerative stem cells; the basal keratinocyte marker CK5, reflecting the basal state of epidermal differentiation; and specific marker for the arrector pili muscle α‐SMA, which is responsible for piloerection. These results demonstrated that all these markers were specifically expressed in their respective structures within the reconstructed HFs, with distribution patterns highly consistent with those in native HFs. Together, these molecular findings indicate that the reconstructed HFs not only achieved complex structural assembly morphologically but, more importantly, attained a mature state comparable to native follicles in terms of protein expression and functional differentiation. In the absence of external interference, the regenerated hairs underwent cyclic growth, and distinct hair shaft structures could still be observed 90 days after HFO implantation (Figure S11). These results provided a solid experimental basis for their further application in regenerative medicine.

Finally, to assess whether the generated HFs possess cycling capability comparable to that of native HFs, a depilation assay was performed on the black hairs constructed via cryoMN@HFO at day 28. As shown in Figure 6d, mature black hairs that had emerged through the skin were cut with tools. By day 42 (two weeks after depilation), most of the cut hair shafts had fully regrown. The presence of normal cuticle layers and other typical morphological characteristics was confirmed by SEM analysis of the regrowing hair shafts, which were similar to the regenerated hair shafts on day 28 (Figure 6e). Further systematic immunofluorescence analysis of the regenerated skin sections for key molecular markers of various HF structures revealed that four representative markers were still specifically expressed in the reconstructed HFs (Figure 6f). In addition to hair clipping, we also conducted studies on hair regeneration from HFOs after plucking. It is found that as long as the HF structure within the skin is not damaged during plucking and only the hair shaft is removed, hair regrowth would occur normally (Figure S12). These results demonstrated that the regrowing HFs have achieved a mature state functionally comparable to that of native follicles, successfully realizing the biomimetic construction of HFs. Moreover, to further confirm biosafety, H&E staining of major organs was conducted on Day 42. The images showed intact organ architecture without significant pathological abnormalities (Figure S13). In all, this study provides an expanded donor source for hair regeneration and establishes a convenient, safe, and stable transplantation strategy for the treatment of hair loss.

## 3 Conclusion

In summary, we have successfully fabricated cryoMN@HFO using a hierarchical microfluidic organoid‐on‐a‐chip platform for biomimetic HF reconstruction. The custom‐designed microfluidic chip, integrating fluidic channel and microneedle template modules, enabled the uniform distribution of embryonic ECs and MCs into needle‐shaped microwells and subsequent in situ generation of morphologically consistent HFOs. Through controlled fluid exchange, a CPA‐containing GelMA pregel solution was infused to encapsulate the HFOs, followed by UV polymerization, gradient freezing, and demolding to obtain the final cryoMN@HFO. The resulting cryoMNs exhibited ideal biocompatibility and robust skin penetration capability, enabling efficient intradermal delivery of organoids. It is notable that a specially designed microcolumn‐involved backing plate served as an anti‐frostbite barrier by minimizing direct skin contact with the frozen substrate. Functional assessments confirmed that the cryoMNs maintained high organoid viability and hair‐inducing potential. In vivo studies showed that both the backing plate and needle tips dissolved after transplantation without causing permanent skin injury, and the delivered HFOs developed into fully functional, structurally mature HFs. These findings demonstrate that the proposed cryoMN@HFO system would offer a promising high‐throughput strategy for functional HF regeneration with the advantage of customizable patterns.

## 4 Experimental Section

### 4.1 Animals

This study utilized a total of ten neonatal C57BL/6J mice, three 5‐week‐old male nude mice, and six 5‐week‐old female nude mice. All animal procedures were approved by the Animal Care and Welfare Committee of Southeast University (No. 20241224001).

### 4.2 Template‐Integrated Microfluidic Chip Construction

A 5 × 5 array positive mold was first designed using SolidWorks software. The array consisted of specially designed protruding structures, each featuring a conical tip with a height of 1200 µm and a base edge diameter of 800 µm, connected to a cylindrical microwell of identical base diameter and a height of 1000 µm. Additionally, a quadrangular prism protrusion with a base side length of 300 µm and a height of 1000 µm was integrated at the center of each 2 × 2 microwell sub‐array (Figure S10). Subsequently, a mixture of PDMS (Dow Corning) and curing agent at a 10:1 ratio was poured into this 3D‐printed positive mold. After vacuum degassing and thermal curing at 75°C, the final PDMS polymer template was obtained by demolding. The PDMS template was then subjected to a 15‐min oxygen plasma treatment, followed by incubation in a 2% (w/v) Pluronic F127 solution for 24 h to reduce cell adhesion, and finally rinsed three times with PBS buffer. For final assembly, the upper PDMS cover and the lower substrate were bonded using a vacuum fixture, and the assembled unit underwent irradiation sterilization to maintain aseptic conditions.

### 4.3 Cell Culture

Following an established protocol provided by Fukuda et al., epithelial cells (ECs) and mesenchymal cells (MCs) were harvested from neonatal C57BL/6J mice and subsequently dissociated [15]. The isolated cells were then resuspended in a Matrigel‐supplemented medium, formulated with Advanced DMEM/F‐12 (Thermo Fisher Scientific), 1% GlutaMAX‐I (Thermo Fisher Scientific), 0.2% Normocin (InvivoGen), and 2% Matrigel (Corning).

### 4.4 Preparation and Evaluation of the HFOs

ECs and MCs were combined and resuspended to a density of 2 × 10^7^ cells/mL. Prior to cell seeding, the microcavities of the chip were sterilized by immersion in 75% ethanol, followed by rinsing with culture medium at 4 °C. The cell suspension was then introduced into the inlet channel until the microcavities were completely filled. After allowing 30 min for cell settlement under static conditions, continuous medium perfusion was initiated at a flow rate of 3 µL/minute for ongoing culture in a cell culture incubator (Figure S11). Notably, under this cell resuspension concentration, we observed that the final number of cells settled within each microneedle cavity reached 40,000 cells.

Over a five‐day culture period, morphological changes of the hair follicle organoids (HFOs) were documented daily using optical microscopy. On day 5, HFO samples were collected for histological analysis. Samples were first washed with PBS and subsequently fixed overnight using a 4% formaldehyde solution. After another three washes with PBS, the samples were dehydrated by immersion in a 30% sucrose solution for an hour. Subsequently, the samples were embedded in Optimal Cutting Temperature (OCT) compound (Sakura Finetek) and sectioned into 8 µm‐thick slices. These sections were subjected to immunofluorescence staining for the following antibody markers: CD34, Ki67, α‐SMA, and CK5 (all purchased from Abcam). Additionally, lipid deposition was assessed by Oil Red O staining according to the manufacturer's protocol (Beyotime).

### 4.5 Fabrication and Characterization of the cryoMN@Hfo

After 5 days of culture, a pre‐gel solution was prepared by mixing 10% (w/v) GelMA (Sigma‐Aldrich) with a commercial cryopreservation agent (CPA, Biosharp, containing 10% DMSO) at a 1:1 volume ratio, supplemented with 1% (v/v) 2‐hydroxy‐2‐methylpropiophenone (Sigma‐Aldrich) as a photoinitiator. Of note, in the final working solution, the final concentration of GelMA is 5% (w/v), and that of DMSO is 5%. This pregel solution was then perfused into the inlet channel of the microfluidic chip at a flow rate of 10 µL/minute to completely replace the existing culture medium. Once the microcavities were filled with the solution, the PDMS cover layer was removed, and the liquid in the basal region was replaced with pure CPA. The pregel solution at the bottom was subsequently crosslinked by exposure to UV light for 30 s. The entire chip was frozen at −20 °C for 4 h, followed by careful detachment of the HFO‐loaded cryomicroneedles (cryoMN@HFO). A stepwise freezing protocol was then implemented, involving storage at ‐80 °C for 4 h and subsequent immersion in liquid nitrogen for an hour. It should be noted that for some morphological characterization experiments (as shown in Figure 3e–g, Figures 4b, S1a and Figure S6), the basal substrate was also fabricated using the GelMA‐cryo hydrogel to form a stable backing plate, thereby maintaining the regular arrangement of the microneedle array and facilitating clear observation of the microneedles and HFO structures. Finally, the morphology of the cryoMNs and their encapsulated HFOs was examined and imaged using both optical microscopy and scanning electron microscopy.

### 4.6 Biosafety Test

To evaluate the biosafety of cryoMNs, both cytocompatibility and hemolysis assays were conducted. For the cytocompatibility assessment, MTT assay and live/dead cell staining were performed. NIH‐3T3 and HaCaT cells were seeded in 48‐well plates containing sterile samples and cultured for a total 3 days. Cellular morphology was observed daily, and cell viability was quantified using the MTT assay. Besides, in the hemolysis test, sterile samples were incubated with an equal volume of 2% (v/v) red blood cell suspension at 4 °C overnight. The hemolysis rate was subsequently calculated by measuring the absorbance of the supernatant according to established methods [39].

### 4.7 Mechanical Property Test

Briefly, the cryoMNs were positioned on a metal plate, and a low‐temperature environment was maintained using a liquid nitrogen cooling system. A flat‐ended stainless steel cylindrical sensor was then advanced at a constant speed of 50 mm/minute, applying a vertical compressive force axially to the microneedle tips. The sensor direction was kept perpendicular to the microneedle surface, oriented toward a top rigid aluminum plate. For comparison, thawed cryoMNs served as the control group. In addition to mechanical characterization, skin penetration capability was assessed in a rodent model. The cryoMNs were applied to the dorsal skin of nude mice. Following application, the tissue was excised, processed through standard steps of embedding, sectioning, and hematoxylin and eosin (H&E) staining to microscopically evaluate the penetration profiles created in the skin.

### 4.8 HFOs' Viability Test

Initially, MCs and/or ECs subjected to programmed freezing were embedded within a GelMA‐cryo hydrogel patch. The cell‐laden hydrogel patches were then transferred to a 24‐well plate and processed sequentially as follows: first, cross‐linked under UV light for 30 s; subsequently, subjected to a gradient freezing process at ‐20 °C for 4 h, followed by ‐80 °C for another 4 h; finally, stored in liquid nitrogen (‐196 °C) for 1 h. After thawing at 37 °C, cell viability in the differen groups was assessed using the Live/Dead Cytotoxicity Kit (Beyotime Biotechnology) according to the manufacturer's instructions, and the survival status of the cells was observed under a fluorescence microscope. Additionally, the viability of cells and HFOs loaded within the cryoMN's tips were evaluated using the same method.

### 4.9 HFOs' Hair‐formation Capacity Test

In vivo HF reconstruction was first performed using an established traditional subcutaneous patch assay method [40]. Specifically, HFOs collected from the microfluidic chip before and after freezing were suspended in an equal volume of culture medium. For each injection, five HFOs were subcutaneously transplanted into nude mice using an 18‐gauge needle syringe. After 28 days post‐transplantation, the injection sites were photographed, and the skin samples were harvested for subsequent analysis.

To further evaluate the hair regenerative capacity of HFOs, an intradermal transplantation assay was conducted. Similarly, pre‐ and post‐freezing HFO samples were collected. Under isoflurane anesthesia, superficial stab wounds were created on the dorsal skin of nude mice using a 20‐gauge ophthalmological V‐Lance knife, followed by the transplantation of one HFO aggregates into each wound site. Topical antibiotic ointment was applied post‐procedure, and wound healing was monitored weekly. After 28 days, nascent hair growth was documented with a digital camera, and the newly formed hair shafts were collected for surface structural analysis by scanning electron microscopy.

### 4.10 In Vivo Hair Regeneration Test

For this study, three 5‐week‐old male and three 5‐week‐old female nude mice were housed under specific pathogen‐free conditions. The prepared cryoMN@HFO patches were implanted into the dorsal skin of anesthetized mice. Gentle pressure was applied until the basal substrate dissolved, ensuring firm adhesion of the microneedles. To track the origin of the cells post‐implantation, HFOs were pre‐labeled with a red fluorescent tracer before being encapsulated within the cryoMNs. In vivo monitoring was performed using a live imaging system following implantation.

The implantation sites were photographed on days 7, 14, 21, and 28 post‐procedure. On day 28, the animals were euthanized, and skin samples were collected for further analysis. The samples underwent post‐fixation, dehydration, and paraffin embedding, followed by sectioning into serial slices. These sections were stained with H&E for general histological evaluation under an upright microscope. Concurrently, immunofluorescence staining was carried out to detect the expression of cell‐specific markers, including α‐SMA, CD34, Ki67, and CK5.

To further assess the hair cycle regeneration capability of the newly formed follicles, hair shafts that had fully penetrated the skin were completely cut using scissors on day 28. Regrowth was documented photographically 14 days after cutting. The corresponding HF samples were then harvested and subjected to the same immunofluorescence staining protocol as described above.

### 4.11 Statistical Analysis

All experiments were performed with a minimum of three independent biological replicates. Data are presented as the mean ± standard deviation (SD). Statistical analysis was carried out using one‐way ANOVA in Origin 2021 software, with significance thresholds defined as follows: not significant (ns, *p* > 0.05), ^*^ *p* ≤ 0.05, ^**^ *p* ≤ 0.01, and ^***^ *p* ≤ 0.001.

### 4.12 Ethics Declaration

All animal procedures were approved by the Animal Care and Welfare Committee of Southeast University (Approved number: 20241224001).

## Author Contributions

**Xinyue Cao**: writing – original draft, methodology. **Dongyu Xu**: writing – review and editing. **Minhui Lu**: writing – review and editing. **Yuanjin Zhao**: conceptualization, funding acquisition.

## Conflicts of Interest

The authors declare no conflict of interest.

## Supporting information




**Supporting File**: advs75961‐sup‐0001‐SuppMat.docx.

## Data Availability

The data that support the findings of this study are available from the corresponding author upon reasonable request.
