# Supplementary material for: Hair Follicle Seedling Cryomicroneedles from Hierarchical Microfluidic Organoid on a Chip
Source: Adv Sci (Weinh). 2026 Jun 4;13(48):e75961. doi: 10.1002/advs.75961 (PMC13336682; doi:10.1002/advs.75961)
Supplement: Supplementary file 1 [file ADVS-13-e75961-s001.docx]

**SI**

**Hair follicle seedling cryomicroneedles from hierarchical microfluidic organoid on a chip**

Xinyue Cao, Dongyu Xu, Minhui Lu, Yuanjin Zhao*

**Figure S1** (a) Photos of anti-frostbite mechanism. Scale bars are 1 mm. Here, the pure CPA in the backing layer was temporarily replaced with GelMA-cryo hydrogel to ensure morphological integrity during demonstration. (b) Contact area between the frozen region and the skin for the anti-frostbite microneedles (with the microcolumn structure) versus conventional microneedles (without the microcolumn structure).

**Figure S2** Diameter distribution of HFOs with different cell amount on Day 5. Scale bars are 200 μm.

**Figure S3** (a) Photographs of formed hair number by HFOs with different cell amount. Scale bars are 200 μm (above) and 2 mm (below). (b, c) Statistical analysis of the formed hair number (b) and efficiency (c).

**Figure S4** Confocal laser microscope image of the mixed ECs (green) and MCs (red). Scale bar is 200 μm.

**Figure S5** Photo and statistical analysis of hemolysis assay.

**Figure S6** SEM images of the cryoMN@HFO. Scale bars are 500 μm. The pure CPA in the backing layer was temporarily replaced with GelMA-cryo hydrogel to ensure morphological integrity during observation.

**Figure S7** Live (green)/dead (red) cell staining of the mixture of MCs and ECs in pure GelMA hydrogel under sequential freezing and thawing.

**Figure S8** Optical and SEM images of *in vitro* cultured HFOs at Day 28. Scale bars are 200 μm.

**Figure S9** Optical image of the hair generated from a single transplanting site. Scale bar is 1 mm.

**Figure S10** Optical images of the skin received blank cryo-microneedle treatment. Scale bars are 2 mm.

**Figure S11** Optical image of the regenerated hair on day 90 post HFO implantation. Scale bar is 2 mm.

**Figure S12** Optical images of the hair regrowth after plucking. Scale bar is 2 mm.

**Figure S13** H&E staining results of major organs (heart, liver, spleen, lung, and kidney) in different groups. “Control” means normal nude mice, and “HFO” means nude mice received HFO implantation.

**Figure S14** Schematic of the specially-designed protrusions structure.

**Figure S15** Photo of the HFO preparation devices.
